# Development of a triage protocol for patients presenting with gastrointestinal hemorrhage: a prospective cohort study

**DOI:** 10.1186/cc6878

**Published:** 2008-04-22

**Authors:** Aneesa M Das, Namita Sood, Katherine Hodgin, Lydia Chang, Shannon S Carson

**Affiliations:** 1 Sleep Institute of Augusta, 3685 Wheeler Road, Suite 101, Augusta, GA 30909, USA; 2 201 Davis Heart and Lung Research Institute, 473 West 12th Avenue, Columbus, OH 43210, USA; 3 University of Colorado Health Sciences Center, 4200 East 9th Avenue, Mailbox C272, Denver, CO 80262, USA; 4 Division of Pulmonary and Critical Care Medicine, 130 Mason Farm Road, 4th Floor Bioinformatics Building, CB# 7020, University of North Carolina at Chapel Hill, NC 27599-7020, USA

## Abstract

**Introduction:**

Many patients presenting with acute gastrointestinal hemorrhage (GIH) are admitted to the intensive care unit (ICU) for monitoring. A simple triage protocol based upon validated risk factors could decrease ICU utilization.

**Methods:**

Records of 188 patients admitted with GIH from the emergency department (ED) were reviewed for BLEED criteria (visualized red blood, systolic blood pressure below 100 mm Hg, elevated prothrombin time [PT], erratic mental status, and unstable comorbid disease) and complication within the first 24 hours of admission. Variables associated with early complication were reassessed in 132 patients prospectively enrolled as a validation cohort. A triage model was developed using significant predictors.

**Results:**

We studied 188 patients in the development set and 132 in the validation set. Red blood (relative risk [RR] 4.53, 95% confidence interval [CI] 2.04, 10.07) and elevated PT (RR 3.27, 95% CI 1.53, 7.01) were significantly associated with complication in the development set. In the validation cohort, the combination of red blood or unstable comorbidity had a sensitivity of 0.73, a specificity of 0.55, a positive predictive value of 0.24, and a negative predictive value of 0.91 for complication within 24 hours. In simulation studies, a triage model using these variables could reduce ICU admissions without increasing the number of complications.

**Conclusion:**

Patients presenting to the ED with GIH who have no evidence of ongoing bleeding or unstable comorbidities are at low risk for complication during hospital admission. A triage model based on these variables should be tested prospectively to optimize critical care resource utilization in this common condition.

## Introduction

Acute gastrointestinal hemorrhage (GIH) can be caused by a wide spectrum of lesions, including those in the upper and lower gastrointestinal tracts. Because acute GIH can be life-threatening in some patients, a large proportion of patients presenting with this condition to US hospitals are admitted and monitored in the intensive care unit (ICU) [1]. ICU admission of these patients can contribute to significant hospital costs [2-4]. However, only 19% to 28% of patients with GIH experience complications that require ICU interventions [5-8]. For the remaining patients, their initial episode of bleeding is self-limited and they are stabilized in the emergency department (ED). Consequently, costly and often scarce ICU resources are used for stable patients. Several studies have shown that there is a great deal of variation between hospitals in the proportion of patients with GIH who are managed in the ICU versus a regular medical or surgical floor [6-9]. It is likely that availability of resources accounts for some of this practice variation, but it remains clear that most physicians are not confident about which patients presenting with GIH can be safely managed without ICU monitoring after stabilization in the ED. Development and implementation of a reliable method to identify patients with acute GIH who are at low risk for early complications would decrease ICU admissions in most hospitals and could improve overall care to critically ill patients by appropriate allocation of resources.

Several investigators have sought to define clinical variables to identify patients with GIH who are at high risk for complication during hospitalization. The most effective approaches involve endoscopic assessments in the ED [10-14]. Immediate endoscopy, however, is not feasible in the ED in most hospitals. Many other approaches are specific for acute upper or acute lower GIH [7,8,15-17], but the source of the bleed is not always known prior to endoscopy. Kollef and colleagues [6,18] identified the BLEED criteria: (a) ongoing *B*leeding, (b) *L*ow systolic blood pressure (BP), (c) *E*levated prothrombin time (PT), (d) *E*rratic mental status, and (e) unstable comorbid *D*isease as risk factors for complication of GIH at any time during hospitalization after an initial 24 hours of stabilization. However, it is not known how well these variables predict the likelihood of complication within the *first 24 hours *after admission from the ED. Determination of *early risk *would be necessary in assessing whether patients should be admitted to an ICU for management and monitoring versus a regular hospital floor.

The purpose of our study was to evaluate variables from the BLEED criteria for their ability to predict complications from GIH within the first 24 hours of hospitalization. A further objective was to define the utility of the predictive variables for use in a functional triage protocol for hospital admission from the ED.

## Materials and methods

This study was conducted in three phases. First, variables were assessed in a retrospective cohort of 188 patients (development set). Second, predictive variables were reassessed using a prospective cohort of 132 patients (validation set). Finally, the utility of the predictive variables for use in a triage model was assessed in a simulation study using the validation cohort which was compared to actual practice during that time period. The study was conducted at the University of North Carolina Hospitals, a 700-bed tertiary care hospital. The University of North Carolina Institutional Review Board reviewed and approved the study protocol.

### Development set

All adult patients admitted to the hospital from the ED for GIH from September 1998 to August 1999 were identified from hospital databases, and their medical records were obtained for data abstraction. Patients were excluded if they were less than 18 years of age, if they were directly admitted from a physician's office or from another hospital, if they had a previous diagnosis of inflammatory bowel disease, or if they were previously enrolled in the study. Data were collected using a uniform data sheet. Two investigators abstracted data, and 10% of charts were reviewed to ensure inter-rater reliability.

### Variables

In addition to patient demographics, the following variables identified in the ED were recorded: presence of chronic disease, presenting symptoms, neurologic dysfunction, comorbidities, heart rate, BP, hematocrit, platelet count, and coagulation studies. After discharge from the ED, admission unit, admitting service, radiographic or endoscopic evaluations, and ICU and hospital outcomes were measured.

### Definitions

Consistent with previous descriptions of the BLEED criteria [6,18], ongoing bleeding in the ED was defined as red blood by emesis or nasogastric (NG) aspirate or hematochezia at the time of evaluation in the ED. Low systolic BP was a systolic BP below 100 mm Hg at any time in the ED. Elevated PT was defined as 1.2 times the upper limit of the normal range. Erratic mental status included syncope, confusion, or coma in the ED. Unstable comorbid disease was defined as any condition other than GIH which would require admission to the ICU.

### Outcomes

A complication was defined as death or rebleeding in the first 24 hours of hospitalization after being admitted from the ED. Rebleeding was defined as documentation of any hematemesis, red blood per NG tube, hematochezia or melena associated with a decrease in hematocrit greater than 6%, or a decrease in systolic BP to less than 90 mm Hg.

### Validation set

All patients consecutively admitted to the hospital from the ED with a diagnosis of GIH between August 2004 and January 2005 were prospectively identified by daily review of ED admissions. Patient records were reviewed for the same predictor variables and outcomes as in the development set, with the addition of Acute Physiology and Chronic Health Evaluation II (APACHE II) scores, which were calculated from the most physiologically abnormal values obtained in the ED [19]. Three investigators abstracted data, and 20% of charts were reviewed to ensure inter-rater reliability.

### Triage simulation

A triage model for guiding admission of patients from the ED to the hospital floor or critical care units was created. Variables that proved to be consistently useful predictors of complications within the first 24 hours of admission in both cohorts were used to designate patients in the validation cohort as critical care admissions or floor admissions. Critical care included either ICU (1:2 nurse-to-patient ratio) or critical care stepdown (1:4 nurse-to-patient ratio). Numbers of patients admitted to critical care or floor care using the predictive variables were compared with actual physician practice during the study periods as a resource utilization analysis, and the incidence of complications occurring on the floor was compared in both groups as a safety analysis.

### Statistical analysis

Descriptive statistics were expressed as mean ± standard deviation, median (interquartile range), or percentage. Associations between BLEED variables, APACHE II score (dichotomized at a score of 15), and the outcome complication within 24 hours of admission were performed using chi-square tests and expressed as risk ratios (RRs) with 95% confidence intervals (CIs). Sensitivity, specificity, positive predictive value, and negative predictive value for each variable and for combinations of variables were calculated from standard 2 × 2 tables, including 95% CIs. When specific variables were not measured, values were considered normal. All statistical analyses were performed with STATA 8.0 software (StataCorp LP, College Station, TX, USA).

## Results

One hundred eighty-eight adults admitted to the University of North Carolina Hospitals from the ED with GIH between September 1998 and August 1999 were identified from hospital databases for the development set. One hundred thirty-two adults were admitted to the University of North Carolina Hospitals from the ED with GIH between August 2004 and January 2005 and followed prospectively for the validation set. The baseline characteristics of both patient groups are shown in Table 1. Patients in the two groups were similar in age and gender, but a higher proportion of patients in the validation set manifested melena or bright red blood per rectum in the ED. For the validation set, patients spent an average of 6.9 ± 3.4 hours in the ED. Nineteen of 22 patients with hematemesis or red blood by NG tube aspiration underwent endoscopy a median of 8.6 (6.8 to 16.3) hours after presentation. Hospital length of stay was 3.5 ± 4.7 days.

**Table 1:** Patient characteristics

	Development set (n = 188)	Validation set (n = 132)
Age, years	64 ± 18	63 ± 16
Gender		
Male	108 (57)	85 (64)
Female	80 (43)	47 (36)
Cirrhosis	19 (10)	21 (16)
End-stage renal disease	9 (5)	10 (8)
Manifestations in emergency department		
Hematemesis	3 (2)	10 (8)
Red blood per nasogastric tube	20 (11)	12 (9)
'Coffee ground' emesis	25 (13)	19 (14)
Red blood per rectum	32 (17)	39 (30)
Melena	17 (9)	22 (17)
None	95 (50)	47 (36)

Values are expressed as mean ± standard deviation or number (percentage).

A total of 23 (12.2%) patients in the development set had complications in the first 24 hours after admission from the ED, and 22 (16.7%) patients in the validation set had complications in the first 24 hours. Three patients from the development set (none in the first 24 hours) and 5 patients from the validation set (2 in the first 24 hours) died during the hospitalization. Only two deaths from the development set and one from the validation set were attributable to complications of GIH.

Table 2 includes results of bivariate analyses of the development set for the association of individual BLEED criteria with the outcome complication within 24 hours. The presence of red blood (hematemesis, red blood per NG tube, or red blood per rectum) and elevated PT were significantly associated with early complication in the development set (RR 4.53, 95% CI 2.04, 10.07, and RR 3.27, 95% CI 1.53, 7.01, respectively). An APACHE II score of greater than 15 was evaluated in the validation set and was not significantly associated with early complication (RR 0.74, 95% CI 0.24 to 2.32; *P *= 0.608).

**Table 2:** Risk of early complication associated with BLEED variables in the development set (n = 188)

	Complication, number (percentage)	No complication, number (percentage)	Risk ratio (95% confidence interval)	*P *value
Red blood				
Present	15 (27)	40 (73)	4.53 (2.04, 10.07)	0.0001
Not present	8 (6)	125 (94)		
Low systolic blood pressure				
Present	3 (13)	20 (87)	1.06 (0.34, 3.28)	0.92
Not present	20 (12)	142 (88)		
Elevated prothrombin time				
Present	7 (33)	14 (67)	3.27 (1.53, 7.01)	0.003
Not present	16 (10)	141 (90)		
Erratic mental status				
Present	3 (16)	16 (84)	1.28 (0.51, 3.22)	0.62
Not present	20 (12)	149 (88)		
Unstable comorbid disease				
Present	2 (17)	10 (83)	1.39 (0.37, 5.23)	0.63
Not present	21 (12)	154 (88)		

BLEED is an acronym for ongoing Bleeding, Low systolic blood pressure, Elevated prothrombin time, Erratic mental status, and unstable comorbid Disease.

Table 3 compares the performance of combinations of the five BLEED criteria for predicting complications within 24 hours for patients in the development and validation sets. The presence of any of the five variables was analyzed for the first analysis. Subsequent analyses included fewer variables in order to reduce the model and improve specificity. The presence of red blood, elevated PT, or unstable comorbidity was analyzed because of the significant association of red blood and elevated PT with early complication and because the presence of an unstable comorbidity along with GIH would likely lead to critical care admission for monitoring regardless of other risk factors. The final model included only red blood or unstable comorbidity, recognizing that a significant number of patients with elevated PT would also present with red blood, and there was likely significant overlap between the two variables. The three combinations performed similarly with regard to sensitivity for both the development and validation sets. Specificity was slightly lower in the validation cohort for each combination. The combination of red blood or unstable comorbidity had the highest specificity compared with the other combinations. The three combinations had similarly high negative predictive values in both cohorts.

**Table 3:** Performance of models in predicting complications within 24 hours of admission from the emergency department

	Sensitivity (95% CI)	Specificity (95% CI)	Positive predictive value (95% CI)	Negative predictive value (95% CI)
Meets at least one BLEED criterion				
Development, n = 96 (51%)	0.83 (0.67, 0.98)	0.53 (0.46, 0.61)	0.20 (0.12, 0.28)	0.96 (0.91, 1.0)
Validation, n = 84 (63%)	0.77 (0.60, 0.95)	0.39 (0.30, 0.48)	0.20 (0.12, 0.29)	0.90 (0.81, 0.98)
Red blood or unstable comorbidity or elevated prothrombin time				
Development, n = 71 (40%)	0.74 (0.56, 0.92)	0.65 (0.57, 0.92)	0.24 (0.14, 0.34)	0.94 (0.90, 0.99)
Validation, n = 71 (55%)	0.73 (0.62, 0.82)	0.48 (0.43, 0.53)	0.23 (0.17, 0.27)	0.89 (0.85, 0.94)
Red blood or unstable comorbidity				
Development, n = 63 (34%)	0.70 (0.51, 0.88)	0.71 (0.64, 0.78)	0.25 (0.15, 0.36)	0.94 (0.90, 0.98)
Validation, n = 66 (50%)	0.73 (0.54, 0.91)	0.55 (0.45, 0.64)	0.24 (0.14, 0.35)	0.91 (0.84, 0.98)

BLEED is an acronym for ongoing Bleeding in the emergency department (red blood per nasogastric tube, hematochezia, or red blood per rectum), Low systolic blood pressure, Elevated prothrombin time, Erratic mental status, and unstable comorbid Disease. CI, confidence interval.

**Figure 1:**
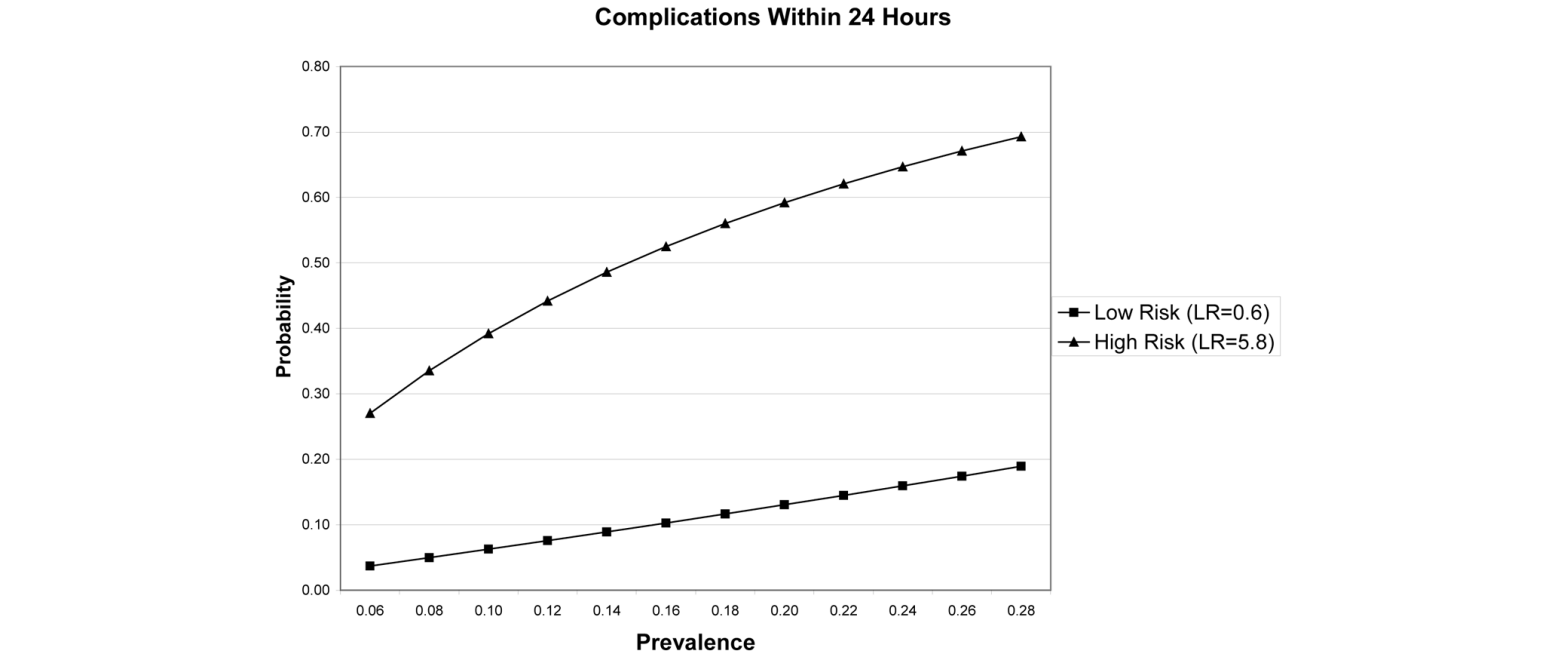
Probability of complication within 24 hours for patients designated as high risk (presence of red blood by emesis or nasogastric aspirate or hematochezia in emergency department and/or unstable comorbidity) or low risk (neither risk factor), plotted by prevalence of complication in a given patient population. LR, likelihood ratio.

Figure 1 uses likelihood ratios to show the probability of complication according to the presence or absence of the risk factors. Risk is plotted relative to prevalence of complications according to published series [5-8]. The low-risk group has no red blood or unstable comorbidity in the ED and the high-risk group has one of these risks present. These risk factors distinguish clearly between high-risk and low-risk groups.

A triage model for admitting patients from the ED to the hospital floor or critical care units was created using combinations of risk factors. The presence of risk factors in the ED would designate a patient as high risk for complication within 24 hours of hospital admission. Based on the model, high-risk patients would be admitted to a critical care floor for monitoring and further management. Low-risk patients could be admitted to the hospital floor. The actual number of patients who were admitted to different levels of care as part of usual care was compared with admission decisions that would have been made using the triage model. The incidence of complications occurring in patients at each level of care was also compared (Table 4). Compared with actual physician practice, a triage model using the presence of any of the BLEED criteria would result in an increase in the number of patients admitted to critical care units (84 versus 76) without any reduction in the number of patients experiencing early complication on the floor. The combination of red blood or unstable comorbidity would result in fewer critical care admissions than usual care (66 versus 76) with no increase in the number of floor patients who experience complications (6 in both models).

**Table 4:** Triage model simulations for validation cohort

	Number	Complication, number (percentage)	No complication, number (percentage)
Usual practice			
Admitted to critical care	76	16 (21)	60 (79)
Admitted to floor	56	6 (11)	50 (89)
Any BLEED criteria			
Admit to critical care	84	17 (20)	67 (80)
Admit to floor	46	5 (11)	41 (89)
Red blood or unstable comorbidity			
Admit to critical care	66	16 (24)	50 (76)
Admit to floor	66	6 (9)	60 (91)

BLEED is an acronym for ongoing Bleeding in the emergency department (red blood per nasogastric tube, hematochezia, or red blood per rectum), Low systolic blood pressure, Elevated prothrombin time, Erratic mental status, and unstable comorbid Disease.

## Discussion

The results of this study indicate that, of the previously published BLEED criteria, ongoing bleeding (as indicated by the presence of red blood in the ED in the form of hematemesis, red blood per NG tube, or hematochezia) and elevated PT are the most strongly associated with complication within the first 24 hours of hospital admission. In the absence of either of these variables, patients who would otherwise not require ICU admission due to other comorbidities could potentially be admitted to a regular hospital or surgical ward for observation and further diagnostic testing. Because of significant overlap between patients with ongoing bleeding and elevated PT, a triage model based upon the presence of ongoing bleeding or unstable comorbidity could result in the fewest critical care admissions without any increase in the number of patients experiencing complications on the medical or surgical ward.

These data indicate that the majority of patients with symptoms of GIH do not have signs of active bleeding when they present to the ED. Furthermore, they are very unlikely to have recurrence of hemorrhage or other complications, especially after resuscitation with intravenous fluids, red blood cell transfusions, and correction of coagulopathy in the ED. Critical care resources should be reserved for patients who need interventions to stop active bleeding or for management of other persistent organ failures that occur as a result of the GIH. This triage model provides an objective measure that could help to identify these two groups of patients. The model is very simple and would be easy to implement in most ED settings.

The primary outcome in the study by Kollef and colleagues that originally defined the BLEED criteria was complication 'occurring after a period of 24 hours of stabilization during which time no evidence of active bleeding was observed' [6]. However, when using risk criteria at the time of hospital admission to decide where a patient should be initially managed, evidence of instability within the *initial *24 hours is most important. It is highly unlikely that a patient monitored in an ICU for 24 hours who has no evidence of instability during that period would remain in the ICU for subsequent days. Therefore, our primary outcome of interest was complication during the first 24 hours of admission from the ED. While our average time in the ED was 6.9 ± 3.4 hours, almost all patients spent at least 2 hours in the ED for resuscitation. Similar to Kollef and colleagues [6,18], we used rebleeding as our primary outcome signifying instability. Rebleeding does not always require ICU care, but bleeding associated with hemodynamic instability or a notable change in hematocrit typically leads to critical care admission in most settings. Our criterion for a significant episode of rebleeding was more stringent than that of Kollef and colleagues. In our study, patients had to demonstrate evidence of rebleeding associated with a systolic BP of less than 90 mm Hg or a decrease in hematocrit of at least 6.0% (as opposed to 3.0% in the study of Kollef and colleagues). We chose this criterion because smaller levels of blood loss could be managed outside of a critical care unit if not associated with hemodynamic compromise, and we wanted to better account for hemodilution that resulted from infusions of crystalloids during resuscitation in the ED.

The triage variables in the study were analyzed in a cohort identified retrospectively and reanalyzed in a subsequent cohort that was identified and followed prospectively. Specificities for the predictive models were lower in the validation cohort, possibly due to more effective detection of complications in patients followed prospectively. However, negative predictive values remained high, and the triage model simulation illustrates that there would be no increase in complications on the floor using this model compared with usual care. Figure 1 illustrates that the triage model distinguishes well between patients at high risk and low risk for complication. When stringent criteria are used to define complication, reflecting complications that would result in ICU admission, the prevalence rate is lower and the resulting number of patients experiencing a serious complication on the floor would be low.

It would be ideal to develop a triage model with both higher sensitivity and higher specificity than usual care to optimize resource utilization and minimize risk. However, it is difficult to achieve both objectives with a simple functional model. Other prediction models for complication from GIH which require calculation of APACHE II scores in the ED have been published [5,20]. In our study, APACHE II was not predictive of complication within the first 24 hours. In addition, reliable calculation of APACHE II scores is likely to be cumbersome in most busy EDs, and the problem of inter-rater reliability would require constant training and retraining of personnel. A shock index (heart rate/systolic BP) has been used in one model to assess risk for active bleeding in patients who required angiograms when bleeding persisted despite endoscopy [21]. In our more heterogeneous cohort, the shock index was not predictive of complication within the first 24 hours. Other variables such as heart rate or initial hemoglobin level did not perform as well as the BLEED criteria. Protocols that involve endoscopy in the ED would be the most effective method of assessment for triage decisions [10,11], but this is not a resource that is available to all EDs, particularly at night.

This study was performed in a single institution with a relatively small sample size and thus may not be able to be generalized to other hospitals. However, this study builds on work by Kollef and colleagues [6] which validated the BLEED criteria in two different teaching hospitals. Interestingly, ongoing bleeding was the only variable that was a significant predictor of complication in both of the hospitals that they studied.

Another limitation is that complications in patients without evidence of ongoing bleeding may have been prevented by being admitted to the ICU rather than the floor. However, patients admitted to the ICU who did not have persistent shock received the same medications and interventions as similar patients admitted to the floor, with the exception of more intense monitoring in the ICU. Therefore, since the rate of rebleeding was very low in these patients, the additional monitoring was of little value considering the resources involved. All patients had access to endoscopy within 24 hours of admission and the only patients who received it emergently were patients demonstrating evidence of ongoing bleeding, a variable that would have resulted in ICU admission anyway. Ultimately, a prospective before-and-after study, or ideally, a randomized controlled trial would be necessary to confirm that a triage model using these variables would effectively reduce the use of critical care resources without compromising patient outcome. These data provide support for the safety and validity of such an intervention.

## Conclusion

Patients presenting to the ED with symptoms of GIH who do not have evidence of ongoing bleeding (hematemesis, red blood per NG tube, or hematochezia) or unstable comorbidities are at low risk for recurrent bleeding and death. A triage protocol based upon these variables may be able to reduce the number of critical care admissions for these patients without increasing the number of complications that occur on hospital wards. The use of objective measures to guide management of critical care beds can maximize the availability of a scarce resource.

## Key messages

• Many patients who present to the emergency department (ED) with symptoms of acute gastrointestinal hemorrhage (GIH) are admitted to the intensive care unit (ICU) for monitoring, but only 12% to 28% of them experience a complication that requires ICU resources for management. This results in the unnecessary utilization of expensive resources for the majority of the patients.

• Patients presenting to the ED with symptoms of GIH who do not have evidence of ongoing bleeding (hematemesis, red blood per nasogastric tube, or hematochezia) or unstable comorbidities are at low risk for serious complication within the first 24 hours of hospital admission.

• A triage protocol based upon these variables should be studied prospectively to determine whether it could reduce the number of critical care admissions for these patients without increasing the number of complications that occur on hospital wards.

## Abbreviations

APACHE II = Acute Physiology and Chronic Health Evaluation II; BLEED = ongoing Bleeding, Low systolic blood pressure, Elevated prothrombin time, Erratic mental status, and unstable comorbid Disease; BP = blood pressure; CI = confidence interval; ED = emergency department; GIH = gastrointestinal hemorrhage; ICU = intensive care unit; NG = nasogastric; PT = prothrombin time; RR = relative risk

## Competing interests

The authors declare that they have no competing interests.

## Authors' contributions

AMD participated in the design of the study, acquisition of data, and data analysis and drafted the manuscript. NS participated in the design of the study, acquisition of data, and data analysis for the development set. KH and LC participated in acquisition of data and in critical review and revision of the manuscript. SSC conceived of the study, participated in its design and coordination and in data analysis, and helped to draft the manuscript. All authors read and approved the final manuscript.
